# A new morphologic classification of the alveolar ridge after distraction osteogenesis in human patients. A 17 years retrospective case series study

**DOI:** 10.4317/medoral.24196

**Published:** 2020-11-28

**Authors:** José Manuel Somoza-Martín, Alba Vázquez-Casal, Mercedes Suárez-Cunqueiro, Abel García-García, Pilar Gándara-Vila, Mario Pérez-Sayáns

**Affiliations:** 1 DDS, PhD; Associate professor. Oral Medicine and Surgery Unit. Faculty of Dentistry, Universidade de Santiago de Compostela, Spain; 2 DDS, Phd Student. Oral Medicine and Surgery Unit. Faculty of Dentistry, Universidade de Santiago de Compostela, Spain; 3 DDS, PhD; Professor. Integrated Adult Clinical Dentistry. Faculty of Dentistry, Universidade de Santiago de Compostela, Spain; 4 MD, PhD; Head of the Oral Medicine. Oral Surgery and Implantology Unit. Faculty of Dentistry, Universidade de Santiago de Compostela, Spain; 5 Instituto de Investigación Sanitaria de Santiago (IDIS), Spain; 6 DDS, PhD; Professor. Oral Medicine, Oral Surgery and Implantology Unit. Faculty of Dentistry, Universidade de Santiago de Compostela, Spain

## Abstract

**Background:**

To perform a morphologic classification based on the results of bone augmentation after a distraction osteogenesis.

**Material and Methods:**

Thirty-four (34) patients (24 women and 10 men; mean age, 47.1 years (SD=9.5); age range, 23 to 62 years) underwent a total of 42 alveolar ridge distractions before the placement of a total of 89 dental implants. Ridge bone morphology was evaluated as the main ordinal variable. Chi-squared, Kruskal-Wallis and ANOVA one-way test were used.

**Results:**

Category I (30.95%): consisted of wide alveolar rim and no bone defects Category II (28.57%): wide alveolar rim, lateral bone surface concavity. Category III (23.81%): narrow alveolar rim, lateral bone surface concavity. Category IV (2.38 %): distraction transport segment forming a bridge, without bone formed beneath and requiring guided bone regeneration. Category V (9.52%): return of the transport segment to its initial position due to the reverse rotation of the distractor screw. Category VI (4.76 %): distraction transport segment completely lost. Subcategory D (28.57%), consisted of lingual deviation of the distraction axis, occurring in any of the categories I to IV. More men (76.9 %) presented with category I (*p*<0.001). The use of the chisel resulted mainly in categories I and II (69.4 %) (*p*<0.001). GBR was only required in 23.1 % of the cases in Category I (*p*=0.011). The bone height achieved decreases as the category increases, due to the accompanying osteogenic limitations (*p*<0.001). The implants placed in category I were longer 11.5 ± 0.9 mm (CI95% 10.9-11.9 mm) compared to those placed in category III with a length of 10.4 ± 1.5 mm (CI95% 9.5-11.4 mm) (*p*=0.035).

**Conclusions:**

The alveolar ridge after distraction osteogenesis could be divided into six morphologic categories which provide a useful basis for decision-making regarding implant placement.

** Key words:**Osteogenesis, distraction, bone lengthening, Ilizarov technique, dental implants.

## Introduction

The application of alveolar bone distraction is used to increase bone to aid with the placement of dental implants (1). It is based on the principles described by Ilizarov and it has been used since the late 20th century (2). In recent years, distraction osteogenesis has become an important technique that is used in bone augmentation techniques before implant placement. However, to date, no general consensus has been reached regarding the precise indications for alveolar distraction within the framework for vertical ridge augmentation (VRA) (3). A recent systematic review and meta-analysis concluded that alveolar distraction has demonstrated accuracy when a greater VRA (greater than 4 mm) is needed (4).

VRA via distraction osteogenesis presents some considerable advantages when compared with other regenerative techniques, such as guided bone regeneration (GBR) or autologous extraoral bone grafts. Numerous advantages have been described, such as the absence of the need for a donor site, therefore reducing morbidity; the simultaneous formation of soft tissues with bone distraction; the low risk of cross infection; and shortened treatment times. (5,6). Nonetheless, this is a technique-sensitive procedure and it is possible that major and/or minor complications may occur, such as the fracture of transport segments, inferior alveolar nerve lesions, graft occlusion, basal bone fracture or an incorrect distraction vector (7).

When expecTable bone gain is not adequate, it is necessary to resort to bone regeneration in order to place the implants, and in other cases, the implant cannot be placed due to failures in the technique (8). In 2004, our team published a preliminary classification identifying four types of alveolar ridge after distraction osteogenesis (9). This classification was based on a small sample with preliminary observations and certain limitations were observed in terms of the clinical situation.

The aim of this paper is to create a morphologic classification of the alveolar bone ridge in order to shed light on the different clinical situations that may arise after VRA via distraction osteogenesis.

Material and methods

This is a 17 year retrospective case series study with an overall sample of 34 consecutive surgical patients receiving vertical alveolar bone distraction in the Unit of Oral Medicine Oral Surgery and Implantology, Faculty of Medicine and Dentistry of the University of Santiago de Compostela (Spain). The surgical procedure was performed from 2000 to 2017. All of the procedures performed in this study were in accordance with the ethical standards of the institutional and research committee and with the 1964 Helsinki declaration and its later amendments or comparable ethical standards. All of the patients gave their written and verbal consent prior to participating in the study and, likewise they gave permission for the results of this research to be published anonymously. This study was approved by the Regional Ethics Committee of Galicia (Ref. 2018/219) and it was developed based on the recommendations set forth in the STROBE guidelines for observational studies (Supplementary file).

Inclusion criteria: patients over 18 years of age, patients who underwent vertical alveolar osteogenic distraction, patients with adequate post-distraction monitoring until the moment of the implant placement.

Exclusion criteria: Patients who smoked more than 10 cigarettes per day, patients with uncontrolled diabetes mellitus, patients with malignant diseases, patients with immunosuppression, and patients with a drug addiction.

After applying the inclusion and exclusion criteria, the sample size consisted of 34 patients (24 women and 10 men; mean age, 47.1 years; SD: 9.5; age range, 23 to 62 years).

- Preoperative study

All of the patients underwent a panoramic radiography and computed tomography in order to plan the surgical procedure. The distraction technique was applied in the following cases: inadequate bone volume for proper implant placement (implants shorter than 6 mm, bone height less than 7 mm), increased prosthetic space, width and height with a minimum of 4 mm allowing for the correct transport segment preparation.

- Distraction procedure

Distractors were placed according to Chin’s procedure (10), firstly an incision was made in the mucosa at the level of the alveolar crest before lifting the vestibular mucoperiosteal flap (maintaining the lingual bone attachment) for distractions in the mandible, and a palate mucoperiosteal flap (maintaining vestibular bone attachment) was used for distractions in the upper jaw. The same surgical technique was used for all patients although new instruments were incorporated over the years to facilitate the ostectomy. The einstruments used to perform the osteotomy and to prepare the bone segment included chisels, saws, discs and piezoelectric instruments.

One week after placement, distraction commenced at 1 mm daily (mandible) and 0.5 mm daily (upper jaw), reaching a final distraction distance of 6.5 ±1.8 mm (mean ±SD). The distractor was then left in place for 12 weeks to ensure bone consolidation and it was then removed for implant placement.

The ridge bone morphology was characterised during implant placement. The outcome of the implant placements (number and type of complications) was subsequently evaluated for all patients. Implants were subjected to a functional load 3 months after placement. All of the implants were evaluated 1 year after placement in order to assess implant mobility, spontaneous or mastication-induced pain, peri-implant inflammatory signs, and marginal bone loss and/or peri-implant radiolucency in radiographs.

**Figure 1:**
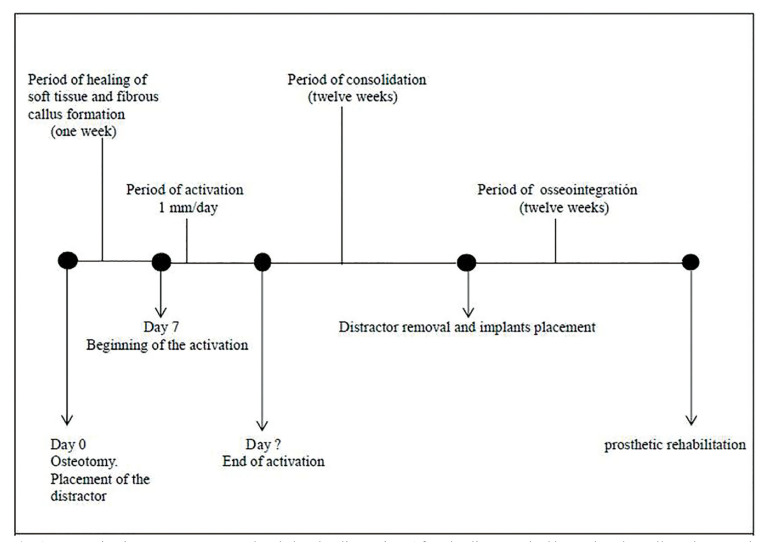
Temporization treatment protocol and alveolar distraction. After the distractor had been placed we allowed one week for the soft tissue to heal before beginning distraction at a rate of 1mm /day. After the distraction, we allowed a 12 week consolidation period before finally placing the dental implants.

The distraction protocol and timing is shown in Fig. 1. With regards to the postoperative instructions, all of the patients were given amoxicillin 500 mg, which they were to take every 8 hours for 7 days (Clamoxyl, GlaxoSmithKline SA, Spain) in addition to a non-steroidal analgesic, ibuprofen (arginine) 600 mg, which they were to take 8 hours for the first 4 days following the surgical procedure (Espidifen, Zambon SAU, Spain). Other postoperative instructions included oral hygiene with 0.12% chlorhexidine solution (Clorhexidine Lacer, Lacer SA, Spain) after brushing their teeth, 3 times a day for 10 days.

- Postdistraction analysis

**Figure 2:**
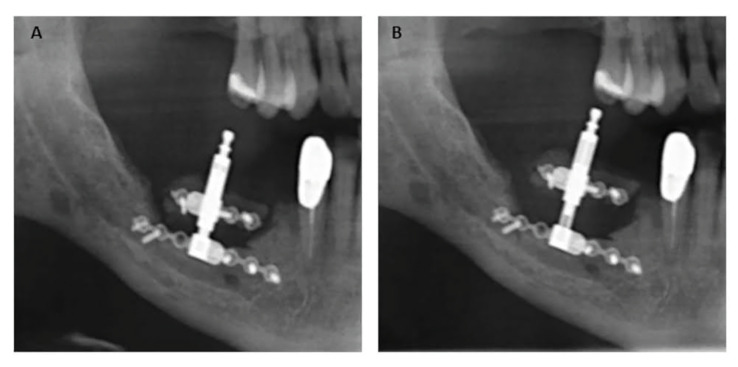
A) Panoramic radiograph taken before the beginning of the distraction period B) Panoramic radiograph taken at the end of the consolidation period. The magnification factor was determined by comparing the size of rods in both panoramic radiographs.

To be able to measure the bone gain we compared the panoramic radiograph that was taken before the distraction with a panoramic radiograph that was taken at the end of the consolidation period. We used the top of the basal plate and the top plate of the osteotomised segment as reference points for measurements. The magnification factor was determined by dividing the actual size of the actuating rod by the size of the actuating rod in the panoramic radiograph (Fig. 2). The final value was obtained by multiplying the obtained measurement by the magnification factor (11). The GBR necessity was evaluated based on the following minimum width requirements: implants ≥ 3 mm with at least 1 mm of bone around the entire implant diameter.

- Study outcomes

The determination of the post-distraction bone category was evaluated in a mixed way: clinically and radiologically. At the clinical level, categories I-IV were evaluated at the time of the implant placement once the distractor had been removed. This evaluation was performed by the oral surgeon, conforming to the characteristics indicated below. Categories V and VI were evaluated radiologically and these were simply verified clinically. All of the radiological measurements were performed once the distraction period had ended and before the distractor had been removed. For the radiological measurements, two operators (AVC and JMSM) were calibrated on a representative sample of 10 radiographs

- Statistical analysis

This study was based on a descriptive analysis and the results have been expressed in mean (standard deviation) and frequencies (percentage) depending on the quantitative and qualitative variables. A statistical analysis was conducted in which the ridge classification was considered as the main ordinal variable. Contingency Tables were then created and the Chi-squared test or Kruskal-Wallis were used to observe the differences in the area, location, type of osteotomy and distractor, and the need for GBR. The ANOVA one way test was used to observe the differences in terms of age, VRA mm and the length of the distracted segment, and the diameter and mean length of the implants placed after distraction. Cohen’s Kappa was used in order to determine the extent of the agreement between both observers. All values of *p* ≤ 0.05 were considered as significant.

## Results

- Distraction osteogenesis

A total of 42 alveolar distractions were performed in 34 patients (40 in the mandible and maxilla 2). 27 patients underwent unilateral distraction; 6 patients underwent bilateral distractions and the remaining patients underwent 3 distractions (bilateral, mandibular and maxilla unilateral). With regards to the location, 4 distractions were performed in the anterior region (3 in the mandible and 1 in the maxilla) and 38 distractions were performed in the posterior region (37 in the mandible and maxilla 1). Only two different types of distractors were used: Modus MDO 1.5/2.0 (Medartis, Basel, Switzerland) was used in 8 distractions, and Lead System (Leibinger, Kalamazoo, USA) was used in 34 distractions. The osteotomy was performed using rotary equipment and chisels in 36 distractions and 6 distractions were performed with piezoelectric surgery (Piezosurgery, Mectron Medical Technology, Carasco, Italy). Distraction failures were reported in only 3% of the cases in which rotary equipment and chisels were used, however 83% of the cases in which piezoelectric surgery was used reported failures.

- Vertical ridge augmentation

The final bone height varied between 0 and 10 mm (average 5.5 mm, SD 2.7 mm). No bone gain was reported in 6 distractions (14.28%) due to a failed technique. The agreement between the observers was determined by the Cohen's kappa index, with a result of 0.82.

- Morphologic bone categories (new proposal)

**Figure 3:**
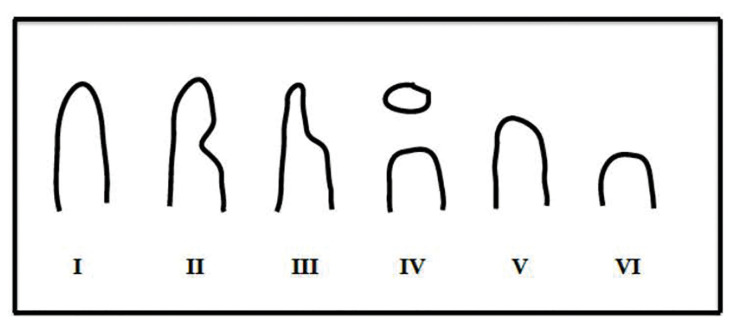
Six morphologic categories were identified (I to VI), with one subcategory (lingual deviation, D) which could potentially arise in any of categories I to IV.

Six morphologic categories were identified (I to VI), with one subcategory (lingual deviation, D), which could potentially arise in any of the categories I to IV. The different morphologies were illustrated diagrammatically (Fig. 3). Ridges assigned to category I showed a wide alveolar rim and no bone defects. Category II showed a wide alveolar rim with lateral bone surface concavity, which was coincident with the incision line.

Ridges assigned to category III showed a narrow alveolar rim (less than 3 mm) with lateral bone surface concavity. In the ridges assigned to category IV the distraction transport segment had formed a bridge without any bone formation beneath that required GBR. In the ridges assigned to category V, the distraction transport segment returned to its initial position during the consolidation period as a result of the reverse rotation of the screw distractor. All of the neoformed bone was lost, and the end result was similar to the initial situation. In the ridges assigned to category VI, the distraction transport segment was completely lost. The final situation included a larger atrophic alveolar ridge than was initially present. In the ridges that were assigned to subcategory D (within any of categories I to IV), the axis of distraction had deviated lingually. In severe cases this required for an osteotomy to be performed in order to free the transport segment and the neoformed bone which is pedicled to the lingual mucosa; the freed segment was then repositioned correctly and fixed to the basal bone by the implant itself.

Table 1 and Table 2 summarise the results. Briefly, category I, 13 distractions (30.95%), category II, 12 distractions (28.57%), category III, 10 distractions (23.81%), category IV, 1 distraction (2.38%), category V, 4 distractions (9.52%) and category VI, 2 distractions (4.76%).

The analytical association data is shown in Table 3. More men presented with category I (76.9 %), with the majority of women presenting with categories II (37.9 %) and III (31 %) respectively (Kruskal-Wallis *p*<0.001). The use of the chisel resulted mainly in categories I and II, representing 69.4 % of the cases, whereas the use of piezosurgery resulted in categories V and VI in 83.3 % of the cases (Kruskal-Wallis *p*<0.001). Categories II and III required GBR in 83.3 % and 80 % of the cases respectively; however, this procedure was only required in 23.1 % of the cases in Category I (Kruskal-Wallis *p*=0.011). Categories IV and VI present in earlier ages, 30 and 29.5 years (SD=3.54) respectively, as opposed to categories I, II and III, at 48.7 years (SD=7.33) (ANOVA *p*=0.034). The number of VDA mm decreases as the category increases, due to the accompanying osteogenic limitations (ANOVA *p*<0.001). With regards to the segment length, the study seems to indicate that the shorter the segment, the higher the category; the mean length of the segment in category I was 27.1 ± 9.3 mm (CI:95% 21.5-32.7 mm), whereas the mean length of the segment in Category VI was 13.0 ± 1.4 mm (CI:95% 0.3-25.7 mm) (ANOVA *p*=0.029).

- Dental implants

Table 1 shows a full description of the dental implants that were placed. The summary of its contents is as follows: after distraction, a total of 89 implants were placed, 84 (94.4 %) in the mandible, 5 (5.6 %) in the maxilla, 11 (12.4 %) in the second or fifth sextant (anterior region) and 78 (87.6 %) in the posterior regions. 75.3% were Straumann (Straumann AG, Switzerland), 10.1% were Frialoc (Friadent GmbH, Germany), 7.9% were Xive (Friadent GmbH, Germany), 5.6 % were NobelReplace (Nobel Biocare, Switzerland), and 1.1% were Frialit-2 (Friadent GmbH, Germany). In 24 (57.1 %) distractions, GBR (guided bone regeneration) was necessary (based on the criteria specified in material and methods section). In these cases a xenograft (Bio-Oss, Geistlich Pharma AG, Switzerland) and a collagen membrane (Bio-Gide, Geistlich Biomateriales, Germany) were used. The mean length was 10.7 ± 1.7mm and the mean diameter was 3.9 ± 0.4mm. The implants placed in category I were longer, and this proved to be statistically significant, 11.5 ± 0.9 mm (CI95% 10.9-11.9 mm) compared to those placed in category III with a length of 10.4 ± 1.5 mm (CI95% 9.5-11.4 mm) (*p*=0.035). No differences were observed in terms of the mean diameter (*p*=0.217).

**Table 1:**
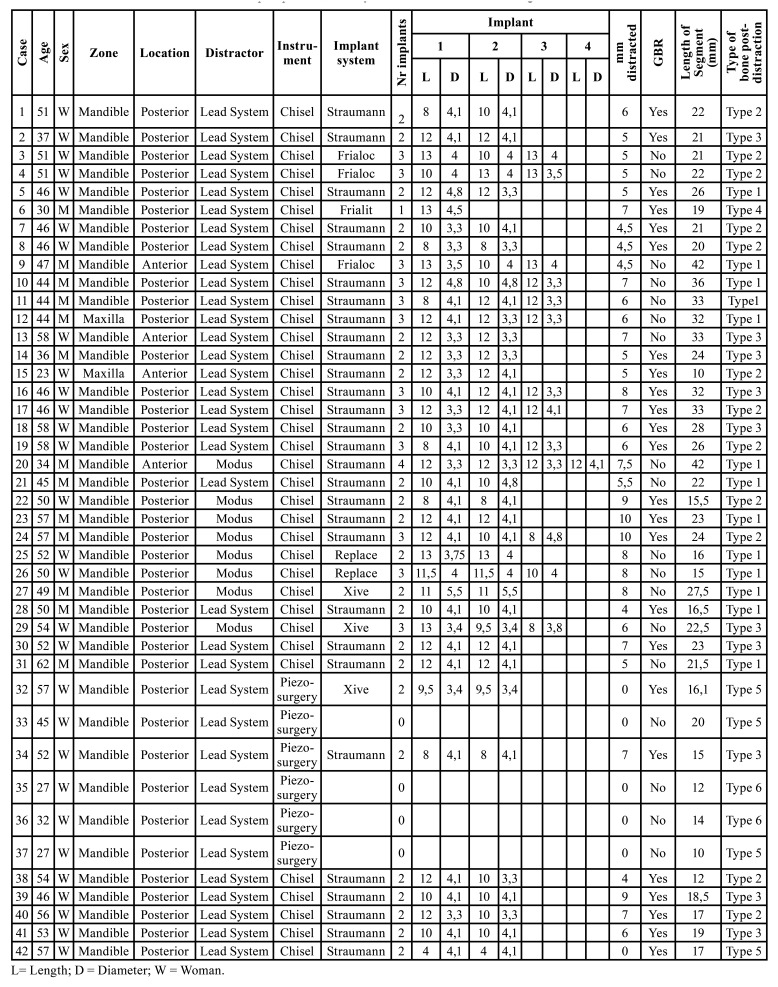
Details and characteristics of the sample specified, case by case. GBR= Guided Bone Regeneration

**Table 2:**
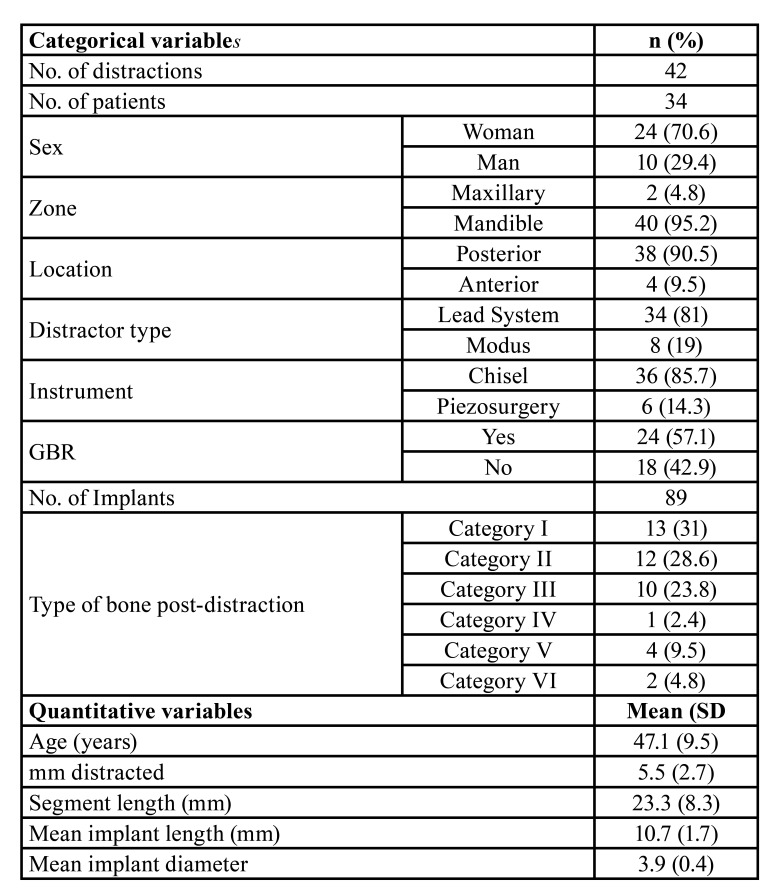
Summary of the main analysed variables.

**Table 3:**
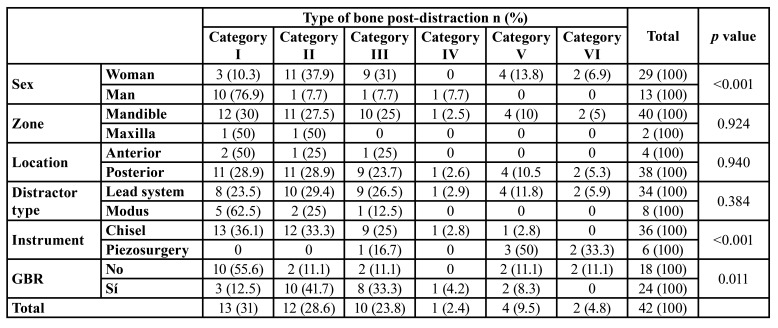
Distribution of categories according to the main variables of the study. *p* value for Kruskal-Wallis test.

## Discussion

Bone formation in distraction osteogenesis is not always spatially uniform and predicTable and there are evident implications for subsequent implant placement (7). In this study, we proposed a modification of the classification published by our research team (9) in which the morphology of the alveolar bone crest post-distraction constitutes a useful basis for making decisions regarding the placement of dental implants. The description of the morphology of the alveolar bone crest was based on observations made during the surgical intervention for the extraction of the distractor and the placement of implants. The authors observed that neoformed bone did not always have the same morphology, but nevertheless, it could always be classified within a reduced number of categories, similar to the bone resorption classifications, which are used in implantology. The area observed during the intervention was the vestibular surface in the mandible and the palatine surface in the maxilla. Our team’s classification had to be changed as a result of the detection of the need for two new categories to be created, this was due to the fact that the apparition of alveolar ridges post-distraction meant that they could not be included in any of the previously described categories. In our sample of patients, Categories IV, V and VI presented a lower casuistry than the other categories, nonetheless they must still be taken into account because of the possibility of their occurrence and because of the possible complications that may arise from them. As this study looks to provide a description of the resulting bone following distraction, we believe it is important that all of the observed bone types are included, regardless of the probability with which they occur. The new classification is more detailed, more objective and more casuistic. However, it is important that the results and the proposed classification are taken with caution in the case of the upper jaw due to the low casuistry, and this data must be confirmed in a longer series.

In this study we used two different distractors, one intraosseous (34 cases) and another juxtaosseous (8 cases), as well as different types of dental implants. Many patients were referred to our service to undergo the alveolar distraction procedure but later the implants were placed by their dentist. However, we believe that the brand of implant used does not condition the described classification, taking into consideration the fact that the bone category post-distraction is established just before the placement of the implants. In this study we analysed the post-distraction bone formation failures according to the instrument that was used to perform the osteotomy. A high percentage of failures were recorded in the cases in which the technique was performed using the piezoelectric instrument and these failures did not occur when rotary instruments and bone chisels were used. We believe that this high failure rate was possibly due to a lack of irrigation in the area during the lingual osteotomy, therefore leading to overheating and resulting in the subsequent resorption of the transport segment. Pavliková *et al*. (12) affirmed that during prolonged or deep cuts, breaks must be taken to prevent overheating, or interrupted cuts should be used. When performing a deep osteotomy it is advisable for a combination of piezoelectric and chisel techniques to be used. However, this finding must be confirmed in another research study with an increased sample size.

We observed that the average bone height obtained (5.5 mm) was lower than the gain observed by other authors (8,13-17). Enislidis *et al* (8) achieved a mean gain of 8.2 mm (5-15 mm), Pérez Sayáns *et al* (11) of 8.36±1.44 mm, Uckan *et al* (14) of 11.6 mm (5-20 mm) and Mazzonetto (17) of 6 mm (0-10.8 mm). These distractions were performed using a saw, chisels and an intraosseous distractor. In another study conducted by Günbay *et al*. (15) a height from 4 to 9 mm was achieved (average 7.8 mm), and the distractions performed with piezoelectric tools and intraosseous, and extraosseous distractors. A recent systematic review performed by Pérez Sayáns *et al* (7) found that the main gain achieved with this distraction technique was 7.55mm. The average gain for extraosseous distractors was 8.13mm and 6.97mm in the case of intraosseous distractors.

According to Pérez Sayáns *et al* (7), by order of frequency, the minor complications were as follows: bad inclination of distraction vector (26.33%); insufficient bone breadth (13.36%); dehiscences (11.83%); paresthesias (8.48%); soft tissue problems (9.66%); pain (4.14%); infection (3.94%); and insufficient height (2.17%). In the present study, GBR was required in 57% of all of the distracted cases. In other studies, such as the one conducted by Mazzonetto *et al*. (18), 38% of patients required autogenous bone graft, of which 81% were placed in the maxillary anterior region, 14% in the posterior mandibular region and 5% in the anterior mandible region. Other authors have verified that in approximately 20% of distractions a new VRA process is required, or alternatively they must resort to other surgical techniques to increase bone. Based on the proposed classification, categories V and VI could be susceptible to retreatment with a new alveolar distraction, although in our study no patient was reoperated (19,20). Urbani *et al*. (21) noted that the cortical bone in the vestibular distraction is thinner than the lingual cortical. Similarly, Oda *et al*. (22) obtained the same result and explained that it was a result of trauma caused during incision and osteotomy. Klug *et al*. (23) described bone defects in nearly 30% of cases that had to be addressed with GBR.

With regards to the categories that were identified in our classification, we observed that category I was the most frequently favourable for the placement of implants. Only 23% of distractions needed GBR. In category II, a lateral concavity that did not reach the crestal edge was observed. This concavity increased the risk of bone defects with fenestration defects being the most frequent complication (9). In the present study 83% of the cases in this category required GBR. In category III, the bony crest was narrow, presenting a concave lateral that reached the crestal edge. The risk of dehiscence defects was greater than in category II (9). In our study, a total of 70% of the cases in this category required GBR. Category IV was an extreme situation, in which a bridge was formed without bone formation between the transport segment and basal bone. Category V was considered as a failure because no bone increase was reported. Despite this failure, four implants were placed because the initial height allowed for the placement of short implants (≤ 6 mm) or for traditional GBR to be performed. The VRA looked to improve the situation from a biomechanical point of view. In category VI, implant placement was not possible, as it produced the complete resorption of the transport segment, and it was observed that the height of the mandibular bone was lower than at the beginning of the intervention. Studies by Nosaka *et al*. (24) and Raghoebar *et al*. (25) considered that both the resorption of the transport segment and the absence of bone formation are caused by the lack of blood supply (25). Wolvius *et al*. also noted that this deficiency is caused by using a bone transport segment that is too small, which also complicates the fixing screw distractor (26). In the present study, the osteotomised bone had a width and a minimum height of 5 mm, which coincides those recorded by most authors (15,27,28).

Concavity of the lateral alveolar crest is a phenomenon called hourglass deformity, which is quite common (18). Several techniques have been proposed to prevent this problem from occurring, including the stimulation of callus and the placement of barrier membranes on the surface of the bone during distraction, with an effect similar to that of GBR.

The main limitations that we have observed during this study were related to the difficulty in correctly monitoring patients that made it difficult for an adequate casuistry to be obtained in this type of research. In many cases dentists refer their patients to our service in order for us to perform the alveolar distraction surgery, but after that the dentists continue with their patients’ treatment and follow-up.

In conclusion, the alveolar ridge can be divided into six morphologic categories after distraction osteogenesis. Category I represents the ideal post-distraction clinical situation. In categories II, III and IV, implant placement is possible with GBR in many cases, and categories V and VI represent a procedure failure. This morphologic classification provides a useful basis for decision-making regarding implant placement.
